# Supplementary figures and images for: Potency of gastrointestinal colonization and virulence of Candida auris in a murine endogenous candidiasis
Source: PLoS One. 2020 Dec 2;15(12):e0243223. doi: 10.1371/journal.pone.0243223 (PMC7710084; doi:10.1371/journal.pone.0243223)

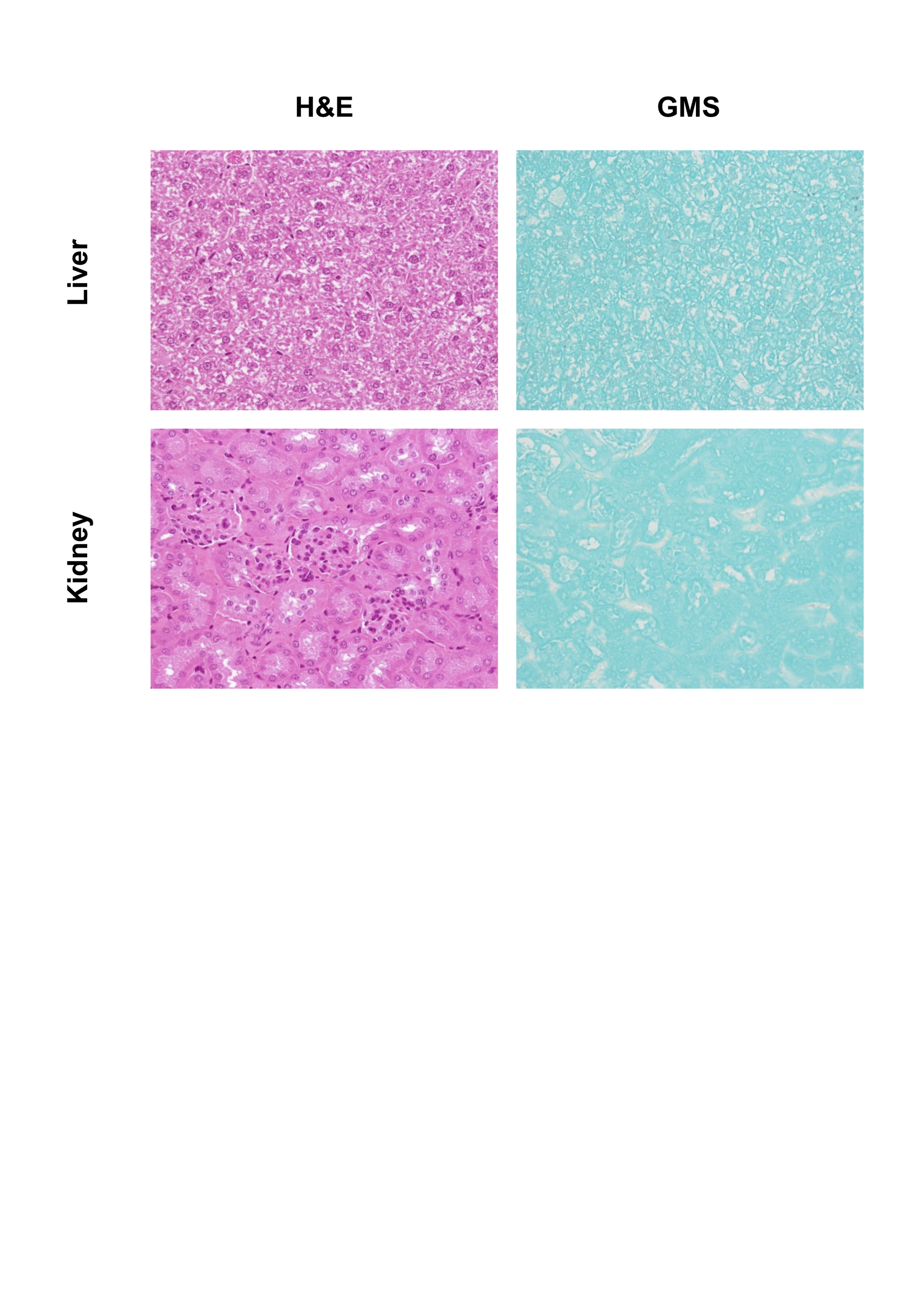

Supplement: S1 Fig — Histopathological analysis of the liver and kidney of mice infected with non-invasive strain JCM 15448, 14 days after inoculation. Hematoxylin and eosin staining (left panels) and GMS staining (right panels) are shown. Original magnification: ×200. (TIF) [file pone.0243223.s001.tif]

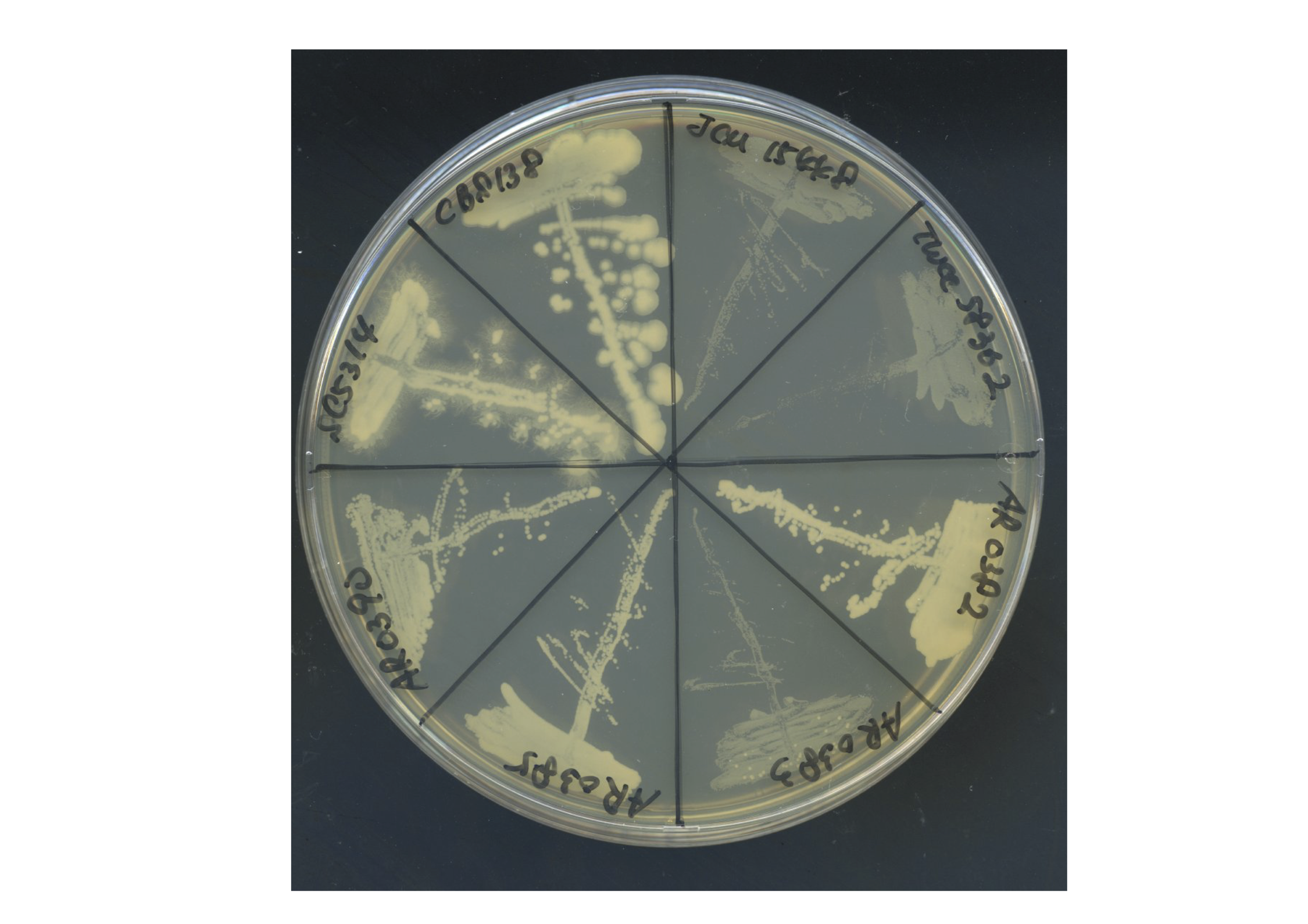

Supplement: S2 Fig — Anaerobic growth of Candida albicans (SC5314), Candida glabrata (CBS 138), and each strain of Candida auris on YPD agar, 7 days after inoculation is shown. (TIF) [file pone.0243223.s002.tif]
