# Supplementary material for: Potency of gastrointestinal colonization and virulence of Candida auris in a murine endogenous candidiasis
Source: PLoS One. 2020 Dec 2;15(12):e0243223. doi: 10.1371/journal.pone.0243223 (PMC7710084; doi:10.1371/journal.pone.0243223)
Supplement: Attachment — Submitted filename: Response to Reviewers.docx [file pone.0243223.s003.docx]

**Response to Reviewer**

**Journal requirements:**

**When submitting your revision, we need you to address these additional requirements.**

**1. Please ensure that your manuscript meets PLOS ONE's style requirements, including those for file naming. The PLOS ONE style templates can be found at https://journals.plos.org/plosone/s/file?id=wjVg/PLOSOne_formatting_sample_main_body.pdf and**

**https://journals.plos.org/plosone/s/file?id=ba62/PLOSOne_formatting_sample_title_authors_affiliations.pdf**

***Response:*** *We modified our manuscript according to the PLOS ONE journal style.*

**2. We note that you have included the phrase “data not shown” in your manuscript. Unfortunately, this does not meet our data sharing requirements. PLOS does not permit references to inaccessible data. We require that authors provide all relevant data within the paper, Supporting Information files, or in an acceptable, public repository. Please add a citation to support this phrase or upload the data that corresponds with these findings to a stable repository (such as Figshare or Dryad) and provide and URLs, DOIs, or accession numbers that may be used to access these data. Or, if the data are not a core part of the research being presented in your study, we ask that you remove the phrase that refers to these data.**

***Response:*** *We appreciate these comments. We modified the manuscript and added two supplementary figure (S Fig 1 and S Fig 2, Line 224 and 363). We also uploaded two supplementary figures.*

**3.PLOS requires an ORCID iD for the corresponding author in Editorial Manager on papers submitted after December 6th, 2016. Please ensure that you have an ORCID iD and that it is validated in Editorial Manager. To do this, go to ‘Update my Information’ (in the upper left-hand corner of the main menu), and click on the Fetch/Validate link next to the ORCID field. This will take you to the ORCID site and allow you to create a new iD or authenticate a pre-existing iD in Editorial Manager. Please see the following video for instructions on linking an ORCID iD to your Editorial Manager account: https://www.youtube.com/watch?v=_xcclfuvtxQ**

***Response:*** *We updated the ORCID ID of corresponding author.*

**4. Please ensure that you refer to Figure 4 in your text as, if accepted, production will need this reference to link the reader to the figure.**

***Response:*** *If accepted, we ensure the production for the reader to link to this figure.*

**Reviewer #1: Overall the paper is clearly presented and the conclusions appear sound based on the available evidence. Minor grammatical corrections are needed, and some methodological clarifications are detailed below.**

***Response:*** *We appreciate the reviewer’s comment. We corrected several grammatical errors and added some methodological clarifications as follows.*

**• Line 54: The authors mention exogenous infection here but doesn’t elaborate on any clinical significance associated with this form of infection.**

***Response:*** *We appreciate the reviewer’s comment. We cited two articles and described the clinical significance of exogenous infection, which was often related with catheter-related bloodstream infections (Line 57-59).*

**• Line 56: An article is needed between “particular” and “gastrointestinal”**

***Response:*** *We appreciate the reviewer’s comment. We added “the previous report describes” between “particular” and “gastrointestinal, and added the reference article (Line 59-61).*

**• Line 125-126: Why were kidney homogenates not plated on YPD agar with antibiotics? What is the rationale behind the different antibiotic conditions for these homogenates?**

***Response:*** *We appreciate the reviewer’s comment. In general, bacteria from gastrointestinal tracts sometimes grow when liver or stool homogenates are plated on YPD agars, on the other hands, bacteria rarely grow when kidney homogenates are plated on YPD agars. Therefore, we empirically used YPD agar with antibiotics for liver or stool homogenates to inhibit bacterial growth, and used YPD agar without antibiotics for kidney homogenates. To mention this point, we added “to inhibit bacterial growth” after penicillin/streptomycin (Line 128).*

**• Line 144: Grammatically this line is difficult to read, specifically “removed for excluding”.**

***Response:*** *We appreciate the reviewer’s comment. As your suggestion, we changed this sentence as “After 4 hours of adhesion, the supernatant of each well was discarded to remove planktonic C. auris cells” (Line 146-147).*

**• Line 158: What physiological signs were used to designate a mouse as moribund?**

***Response:*** *We appreciate the reviewer’s comment. We added signs of moribund (reduced activity, tachypnea, and poorly responsive to external stimuli: Line 161-162).*

**• Line 241-243: Is there anything about AR0390 that suggests why it produced biofilms at a substantially lower level than AR0382?**

***Response:*** *We appreciate the reviewer’s comment. Considering your suggestion, we searched for articles describing these differences, however, we could not find any articles referring to this point. We assume that these biofilm-forming capacities depend on strains, not on clades, however, further investigation is necessary to clarify this point. To mention this point, we added several sentences to the Discussion part (Line 315-317).*

**• Line 259: Grammatically change “All of mice” to “All of the mice”.**

***Response:*** *We appreciate the reviewer’s comment. As your suggestion, we changed “All of mice” to “All of the mice” (Line 269).*
